# Time to endoscopic intervention in patients with upper gastrointestinal patients can be improved with pathway provision

**DOI:** 10.1186/s12885-017-3335-0

**Published:** 2017-05-25

**Authors:** R Singer, P Campbell, C Fernandes, P Statham, D Hochhauser, J Bridgewater

**Affiliations:** 1 University College Hospital, 235 Euston Road, London, UK; 2 UCL Cancer Institute, London, UK

**Keywords:** Gastrointestinal oncology, Endoscopic procedures, Waiting times, Audit cycle

## Abstract

**Background:**

Patients with upper gastrointestinal malignancy often require admission to hospital with dysphagia or jaundice requiring therapeutic endoscopy. Endoscopic intervention is often effective permitting rapid discharge. An efficient service would permit rapid discharge for patients who are often at the end of life. We noted that a majority of patients in hospital under the gastroenterological oncology were admitted with symptoms requiring therapeutic endoscopy.

**Methods:**

We conducted an audit cycle of the inpatient days before and after pathway implementation.

A wait of 1 day was set as acceptable for patients with bleeding as defined by NICE guidance and we set an arbitrary standard of 2 days for patients without bleeding but requiring therapeutic endoscopy. Between the audit cycles, a pathway was built to accommodate these patients.

**Results:**

Inpatient waits improved from a median of 3 days to 1 day. There was no difference in outcome between those presenting with bleeding and other symptoms or any difference in patients requiring different procedures.

**Conclusions:**

Waiting times for endoscopy can be improved with the introduction of a targeted pathway of cancer patients. Further issues including cost, quality of life and nutrition require further intervention.

**Electronic supplementary material:**

The online version of this article (doi:10.1186/s12885-017-3335-0) contains supplementary material, which is available to authorized users.

## Background

Urgent therapeutic endoscopy is commonly needed in patients with upper gastrointestinal (UGI) cancer. Obstruction (oesophageal, gastric, duodenal and biliary) and iatrogenic complications, particularly bleeding from anti-inflammatory and anti-emetic medication are common. Most of these patients are treated with palliative intent and prompt intervention would seem essential. Commonly these procedures comprise oesophagoduodenoscopy (OGD), endoscopic retrogradepancreatography (ERCP) or another procedure such as percutaneous endoscopic gastrostomy (PEG).

In reality, delays in therapeutic endoscopy are common, frustrating and potentially harmful for the patient. While waiting for a procedure, infection, thrombo-embolism and iatrogenic or hospital acquired diarrheoa occur commonly and weight loss while the patient remains nil-by-mouth, commonly on more than 1 occasion, may be significant. There is a self-evident impact on the quality of life patients with a terminal cancer diagnosis having to wait in hospital for procedures. For some cancer patients this time may represent a significant proportion of their survival. Additionally, delays significantly add to hospital costs through prolonged inpatient stays. Critically, endoscopic therapy is often immediately effective and leads to prompt discharge.

NICE guidelines state that unstable patients with severe acute UGI bleeding should be offered endoscopy immediately after resuscitation and that all other patients with upper GI bleeding should be offered endoscopy within 24 h [1]. Cancer patients are a particularly high risk group, with an odds ratio for mortality from upper GI bleeding of 3.8 compared to patients with no co-morbidities.

There are no guidelines on the optimal timing of endoscopy for suspected GI obstruction. There have been several studies of endoscopic intervention for malignant obstruction, but these do not record waiting times for procedures. Additionally the 2012 Cochrane review on interventions for dysphagia in oesophageal cancer found that there was no evidence base to recommend the appropriate timing of self-expanding metal stents [2].

We performed a completed audited cycle of local practice for cancer patients having upper GI endoscopy in a central London teaching hospital practice and describe the outcome following targeted intervention.

## Methods

The audit cycle was performed at a central London teaching hospital. All patients were in specialist oncology beds and the gastroenterology team were contacted to consult.

We searched electronic ward archives to identify patients admitted under the UGI oncology team having an endoscopy. The initial audit period was 20/11/10 to 31/07/11, and the re-audit period 01/01/2014 to 31/07/2014. Endoscopy reports including date of procedure were recovered from the electronic patient record system. At the time of the initial audit endoscopy referrals were not recorded, so waiting time was calculated from the date when endoscopy request was first documented in the electronic ward archive. At the time of the re-audit endoscopy referrals were recorded, so the date of referral was taken from endoscopy records. For patients with suspected UGI bleeding we used an audit standard of 1 day to wait for an OGD, as per the NICE guidelines.

For patients with non-bleeding indications for endoscopy there is no national standard for waiting time. For cost analysis purposes we set a ‘reasonable waiting time’ for inpatient endoscopy for non-bleeding patients of 2 days, based on consensus opinion of the investigators. For patients identified as outliers in the data set, the paper notes were recovered from patient records and reviewed to identify reasons for delays.

Median waiting times were compared with the Mann Whitney U test. Demographic differences were compared with the chi-squared test. Rates of compliance with the audit standard as well as differences in endoscopies carried out within a specified time period were compared with Fischer’s Exact Test.

As part of the audit cycle, following the review of findings from the preliminary audit and prior to the re-audit, a number of recommendations were proposed and implemented. Firstly, a visual pathway tool was developed with the intention of providing a leaner process and greater clarity for teams involved in the referral procedure. Depending on patient eligibility, this also included a day case option for the endoscopic procedure. (prior to this, such patients were routinely admitted) .The pathways (Additional file 1: Figure S1 and Additional file 2: Figure S2) were devised following a scoping exercise to review existing practice involving medical, nursing and administrative staff from both oncology and gastroenterology specialties. The wider team were also consulted during the drafting of the new pathway. To launch and facilitate the smooth initiation of the pathway, copies of the template were disseminated and a series of teaching sessions were carried out. Secondly, a standard time from request to endoscopic procedure for oncology ‘non-bleeding’ cases was agreed at two working days and thirdly a standard time from request to patient review by the gastroenterology medical team for oncology inpatients was agreed at 24 h.

This was an audit therefore no ethics approval was required.

## Results

For the initial audit period and re-audit, we identified 30 and 42 patients respectively who had inpatient endoscopy during the audit periods. In both samples, oesophageal cancer was the most common primary, obstructive symptoms the most common indication. The variation in primary cancer diagnosis was wider in the initial audit, with three primaries that were not seen in the re-audit period: cholangiocarcinoma, duodenal cancer and cancer of unknown primary, however there were no significant statistically differences in primary tumour site between initial audit and re-audit groups. There were also no significant statistically differences in indication for endoscopy within the two groups. Table 1 summarises the patients by cancer diagnosis and indication for the procedure.

**Table 1:** Initial audit and re-audit groups by wait times for endoscopy as well as diagnosis and indication for endoscopy (‘Other’ indications were abdominal pain, broncho-oesophageal fistula, acute diarrhoea, abdominal pain, and one documented only as ‘diagnostic’)

	Initial audit	Re-audit	*P* value
Total no. Endoscopies	30	42	
Median wait (days)	3	1	0.010
IQR (days)	3.8	3.5	
% done within 24 h	26.7	57.1	0.016
% done within 48 h	43.3	61.9	0.096
Tumour site	*N* (%)	*N* (%)	
CUP	2 (6.7)	0	0.06
Cholangiocarcinoma	3 (10)	0	
Colorectal	1 (3.3)	3 (7.1)	
Duodenal	2 (6.7)	0	
Gastric	4 (13.3)	5 (11.9)	
GOJ	2 (6.7)	5 (11.9)	
Oesophageal	16 (53.3)	26 (61.9)	
Pancreas	0	3 (7.1)	
Indication (%)	*N* (%)	*N* (%)	
Bleeding	9 (30)	8 (19)	0.33
Obstructive symptoms	19 (63.3)	33 (78.6)	
Other	2 (6.7)	1 (2.4)	

*CUP* – cancer of unknown primary, *GOJ* – gastro-oesophageal junction

The median wait time for endoscopy was significantly less in the re-audit group, with median wait times falling from 3 days (IQR 1.25–5 days) to 1 day (IQR 1–4.5 days; *p* = 0.010) (Table 1 and Fig. 1). There were also significantly more patients having their endoscopy performed within 24 h in the re-audit group (26.7% vs. 57.1%; *p* = 0.16).

In the initial audit group there was clear pattern for waiting time differences between patients with different primary cancer sites (Table 2). Though those with cholangiocarcinoma appeared to have shorter waiting times this was no significant when compared to the rest of the cohort (*p* = 0.11). Similarly there were no significant differences in wait times between those with oesophagogastric cancer (the largest groups in the initial audit) compared with non-oesophagogastric cancer (*p* = 0.62 and 0.80 respectively). In the re-audit group there were also no clear differences in wait times between patients with different primary cancer sites. When comparing wait times in those with gastric, GOJ and oesophageal cancer to the rest of the re-audit cohort no significant differences were found (*p* = 0.065, 0.79 and 0.32 respectively).

**Table 2:** Median wait times for endoscopy pre and post intervention stratified by primary tumour site and indication for endoscopy

	Median wait times (days)		*p*-value
	Pre-intervention	Post-intervention	
Tumour site			
Gastric	4.0	1.0	0.029
Oesophageal	3.5	1.5	0.074
GOJ	4.5	1.0	0.23
CUP	4.5	-	
Colorectal	2.0	2.0	
Cholangiocarcinoma	1.0	-	
Duodenal	4.0	-	
Pancreas	-	1.0	
Indication			
Bleeding	2.0	1.0	0.04
Obstructive symptoms	3.0	1.0	0.051
Other	8.5	5	

*GOJ* – gastro-oesophageal junction, *CUP* – cancer of unknown primary

When comparing median wait times by primary cancer site between the initial and re-audit groups a significant reduction in wait times in those with gastric cancer was found (median wait 4 vs. 1 days; *p* = 0.029). There was also a trend for reduced wait times in both patients with oesophageal and GOJ cancer but these did not reach significance (*p* = 0.074 and 0.23 respectively, Table 2).

In the initial audit period there were no significant difference between those who endoscopies were performed for bleeding issues compared to those who did not have bleeding problems (median wait 2 vs 3 days; *p* = 0.35). In the re-audit group there was more of a trend for patients with bleeding symptoms to have their endoscopies performed more quickly compared to the rest of the group (1 vs 1.5 days) but these results were not significant (*p* = 0.09).

Comparing wait times between the audit groups a significant reduction in wait times was seen in those with bleeding symptoms (median wait 2 vs 1 days, *p* = 0.04). A similar trend was also seen in those with obstructive symptoms all this did not quite reach significance (*p* = 0.051, Table 2). There was no clear pattern for differences in waiting times by primary cancer or procedure type.

For patients with suspected GI bleeding, compliance with the audit standard was higher in the re-audit group than the initial audit (88% vs 44% respectively), though this did not reach statistical significance (*p* = 0.13). For patients with obstructive symptoms significantly more patients had their endoscopies within 24 h in the re-audit group compared to the initial group (51.6% vs. 21.1%; *p* = 0.042). However there was no significant increase in the percentage patients having their endoscopies for obstructive symptoms by 48 h post request (60.6% vs 42.1%; *p* = 0.25).

### Cost implications

The percentage of patients having endoscopy (for any indication) beyond the reasonable waiting time of 48 h was 58% in the initial audit and 45% in the re-audit, with a total cumulative number of days waited beyond 48 h of 84 and 64 respectively. With a cost of each bed day in our oncology wards of £325, the extra waiting times accounted for £27,300 in the initial audit and £20,800 in the re-audit period. When adjusted for the differences in sample size, the money lost to extra waiting was £9374 lower in the re-audit period or approximately £120/month. This does not include additional costs of end of life inpatient care.

### Outliers

In order to try and describe common reasons for longer delays, we reviewed the notes of the 5 patients who waited longest (Additional file 3: Table S1). The recurring themes for all 5 patients were a lack of resource in gastroenterology department, and communication issues between oncology and gastroenterology. Some patients waited over a week for an individual consultant to place a feeding tube. Outliers were fewer in the re-audit. One patient with UGI bleed (oesophageal cancer) waited for 6 days. The patient had malaena and coffee ground vomiting post oesophageal stent insertion but the haemoglobin was stable and the gastroenterology team felt the patient did not need an urgent OGD. One patient waited 14 days with obstructive symptoms (oesophageal cancer) but during this period was unwell on intensive care.

## Discussion

We performed a retrospective audit of endoscopy procedures in oncology patients in a London teaching hospital. The median waiting time was improved from a median of 3 days to 1 day. This was achieved through the establishment of pathways for the common presentations and increased awareness amongst the team coordinating therapeutic endoscopy.

The impact of waiting for procedures as an impatient is self-evident and only partly quantifiable. If we consider that our population was being treated with palliative intent, it would be reasonable to consider their management as end of life care. Because of the efficacy of endoscopic intervention, prompt discharge would have been anticipated. End of life care in hospital is more costly than community management [3, 4] but we did not formally evaluate this. Less quantifiable outcomes include the impact on quality of life (QL) on cancer patients. Data from patients with an UGI bleed suggest a significant impact on QL [5]. Subsequent studies should aim to evaluate QL and the cost-economic impact in greater detail.

Our audit assessed oncology patients admitted under their oncology team in a cancer centre. For patients who are admitted to a cancer unit, the pathway may differ, with admission under the care of the acute oncology service or the gastroenterological service. We are not aware that this audit has been performed for patients admitted under a different model. As such, the key aspects of the pathway introduction was education and experience of using the pathway. Several educational events were given and the policy widely circulated amongst key staff including clinical nurse specialists, appointments clerks and endoscopists. The process became standard shortly after introduction.

The sustainability of improvement following audit has been identified as an issue [6]. The improvements in our service were established by education and introduction of a defined identity and pathway for cancer patients requiring therapeutic endoscopy. The longevity of this improvement will have to be determined by future audits.

The strengths of our audit were the simplicity and re-audit after intervention. The weaknesses include a lack of depth of data and the relatively small sample size. We were not able to examine whether pathway implementation resulted in fewer admissions. Further issues with respect to the costs, the quality of life and the nutritional status of our patients have been identified and merit further investigation.

## Conclusions

We conducted an audit cycle to determine the effect of a specific pathway for cancer patients waiting for therapeutic endoscopy as inpatients. An improvement in outcome was found suggesting that identification of cancer patients requiring therapeutic endoscopy may be helpful.

## Additional files

Additional file 1: Figure S1. ERCP pathway. (DOCM 39 kb)

Additional file 2: Figure S2. Endoscopy pathway. (DOC 80 kb)

Additional file 3: Table S1. Analysis of outliers. (DOCX 20.9 kb)

## Abbreviations

ERCP: Endoscopic retrogradepancreatography
NICE: National Institute of Clinical Excellence
OGD: Oesophagoduodenoscopy
PEG: percutaneous endoscopic gastrostomy
UGI: Upper gastrointestinal
