# Supplementary material for: Time to endoscopic intervention in patients with upper gastrointestinal patients can be improved with pathway provision
Source: BMC Cancer. 2017 May 25;17:365. doi: 10.1186/s12885-017-3335-0 (PMC5445365; doi:10.1186/s12885-017-3335-0)
Supplement: Supplementary file 1 — ERCP pathway. (DOCM 39 kb) [file 12885_2017_3335_MOESM1_ESM.docm]

# *Patient care pathway for cancer patients requiring an urgent ERCP for biliary decompression*

| **pre-procedure** | 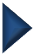 | - During the clinical review the decision to carry out an ERCP (endoscopic retrograde cholangiopancreatogram) procedure will be discussed with the patient. (This may be during clinic, A&E or Supportive Care if the patient has presented as an emergency or on ward) - The clinician will ensure that pre-procedure bloods are taken. (Satisfactory results taken within a week may be used):   - FBC, Us & Es, LFTs, CRP , Clotting   - If not already available, the clinician will ensure that pre procedure imaging is booked:   - In those patients with fully staged malignancy, ultrasound may be suitable for patients with known low bile duct obstruction. All other patients require a pancreatic protocol CT. (Imaging taken within 10 days covering the patient’s current clinical episode may be used)   - In those patients without fully staged malignancy, staging pancreatic-protocol CT chest abdomen, pelvis. - Clinician will contact the Hepatopancreatobiliary (HPB) doctor via mobile (Tel: 07939204237) to confirm ERCP request and if required, to arrange a medical review for patients who are admitted for the procedure - Clinician will download and complete the electronic UCH Pancreaticobiliary new patient referral form located at: http://www.uclh.nhs.uk/ERCP. Completed forms will be emailed to [pancmed@uclh.nhs.uk](mailto:pancmed@uclh.nhs.uk) - If not already aware, the clinician will inform the HPB clinical nurse specialist (CNS) of the ERCP booking - If the clinician assesses the patient to be jaundiced with no evidence of infection and there is not a clinical need for hospital admission, the patient will follow the day case pathway. If there is clinical evidence of biliary sepsis due to bile duct obstruction, the patient will follow the inpatient pathway - HPB CNS will act as Key worker for both Day Case and Inpatient pathways | |
| --- | --- | --- | --- |
| **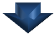** |  | 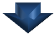 | |
| **DAY CASE PATHWAY** | | | **INPATIENT PATHWAY** |
| - Clinician will provide the patient with the relevant procedure information leaflets - Patient will be given a follow up appointment for the Cancer GI clinic up to a week post procedure - Endoscopy admin team or HPB medical team will contact the patient to advise them of appointment date and of any pre-procedure instructions. (Depending on the clinical case, for most patients this will be within four days) | | | - The clinician will contact the oncology bed manager (bleep 2292) to discuss whether an immediate inpatient admission or a priority bed for the following day is required - If a bed is immediately available, the patient will attend ward as instructed by the oncology bed manager. If the bed is not immediately available, the patient will be referred to the Supportive Care Unit where they will be monitored until bed is ready - Where an in-patient assessment by HPB medical team is requested, this will happen within 18-24 hours of the request. Requests to be made via HPB FY2 (bleep 2060), SpR (via switch), or fellow (bile phone 07939204237) - Ward staff will provide the patient with the relevant procedure information leaflets - HPB team will contact ward to confirm date and time of procedure |
| **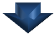 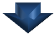** | | | |
| **day of**  **procedure** | 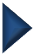 | - Patient will report to the Endoscopy Unit reception at appointment time - Patient will be consented for the procedure - Patient’s baseline vital signs will be recorded - ERCP procedure will be performed - Patient will be transferred to Endoscopy recovery for further assessment and monitoring - While in recovery, the patient will be assessed according to the Post Sedation / Anaesthesia Discharge Scoring System (PADDS). If an inpatient, the patient will be transferred back to their ward when assessed as clinically stable. If a day case, a PADDS score of nine or above must be achieved before they are discharged home - Endoscopy doctor will complete procedure report; a copy will be filed in the patient’s medical notes and will also be available on CDR system - If appropriate, a procedure after care leaflet will be given to the patient | |
| **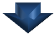** |  | 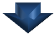 | |
| **discharge & follow up care** | 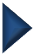 | - Hospital contact numbers will be given to the patient - Prior to discharge, the Endoscopy team will confirm that day case patients have a post procedure follow up clinic appointment with their oncology team | |
