# Supplementary material for: Time to endoscopic intervention in patients with upper gastrointestinal patients can be improved with pathway provision
Source: BMC Cancer. 2017 May 25;17:365. doi: 10.1186/s12885-017-3335-0 (PMC5445365; doi:10.1186/s12885-017-3335-0)
Supplement: Supplementary file 2 — Endoscopy pathway. (DOC 80 kb) [file 12885_2017_3335_MOESM2_ESM.doc]

# *Patient care pathway for cancer patients undergoing an urgent endoscopy procedure including stent, laser, dilatation and bleeds within the inpatient and day case setting*

| **pre- procedure** | 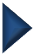 | - During the clinical review the decision to carry out an endoscopy procedure will be discussed with the patient. (This may be during clinic or within A & E or Supportive Care if the patient has presented as an emergency) - If the clinician considers the case to be urgent (required within 24-48 hours) and a standard endoscopy / OGD (Oesophago-gastro-duodenoscopy)/ is required, the patient will follow the day case pathway below. The inpatient pathway will be followed if there is a clinical need for the procedure to be carried out within the inpatient setting | |
| --- | --- | --- | --- |
| **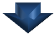** |  | 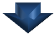 | |
| **DAY CASE PATHWAY** | | | **INPATIENT PATHWAY** |
| - Clinician will contact the Luminal Gastro SpR via mobile or bleep (both numbers available via switchboard) - For upper GI cancer patients, clinician will contact Dr. R.Haidry or Dr. M. Banks ONLY if they fail to contact the Luminal Gastro SpR (contact numbers available via switchboard) - Clinician will complete electronic endoscopy referral form - Clinician will provide the patient with the relevant information leaflets - Patient will be given a post procedure follow up appointment for the Cancer GI clinic - Endoscopy admin staff will contact the patient and advise them of appointment date and of any pre-procedure instructions. (This will be within 48 hours of request for urgent cases) | | | - If an urgent request, the clinician will contact the oncology bed manger on bleep 2292 to discuss whether an immediate inpatient admission or a priority bed for the following day is required - Clinician will document the type of endoscopic procedure required and state whether the patient should be reviewed by the Gastroenterology team within the patient’s medical notes - If a bed is immediately available, the patient will attend ward as instructed by the oncology bed manager. If the bed is not immediately available, the patient will be referred to the Supportive Care Unit where they will be monitored until bed is ready - If not already aware, the clinical team will inform the patient’s clinical nurse specialist (CNS) of their admission - If a request for a Gastroenterology medical review has been made, the oncology SHO will call the Luminal Gastro SpR via mobile or bleep (both numbers available via switchboard). The patient will be reviewed by the Gastro team within 18-24 hours of this request - The ward SHO will complete the electronic endoscopy request form; he/she will contact the Luminal Gastro SpR to discuss urgency of case if required - The Gastro registrar will review all endoscopy requests each week day morning and prioritise - Endoscopy booking staff will contact ward to inform of endoscopy appointment which will be within 48 hours of request |
| **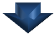** 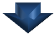 | | | |
| **day of procedure** | 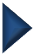 | - Patient will report to the Endoscopy Unit reception at appointment time - Patient will be consented for the procedure - Patient’s baseline vital signs will be recorded - Endoscopic procedure will be performed - Patient will be transferred to Endoscopy recovery for further monitoring and assessment - Endoscopy doctor will complete procedure report; a copy will be filed in the patient’s medical notes and will also be available on CDR system (Clinical Data Repository) | |
| **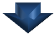** |  | 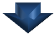 | |
| **discharge & follow up care** | 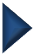 | - While in recovery, the patient will be assessed using the Post Sedation / Anaesthesia Discharge Scoring System (PADDS). If an inpatient, the patient will be transferred back to their ward when assessed as clinically stable. If a day case, a PADDS score of 9 or above must be achieved before they are discharged home - If appropriate, a procedure after care leaflet will be given to patient - Hospital contact numbers will be given to patient - Prior to discharge, the Endoscopy team will confirm that day case patients have a post procedure follow up clinic appointment with their oncology team | |
