# Supplementary material for: Time to endoscopic intervention in patients with upper gastrointestinal patients can be improved with pathway provision
Source: BMC Cancer. 2017 May 25;17:365. doi: 10.1186/s12885-017-3335-0 (PMC5445365; doi:10.1186/s12885-017-3335-0)
Supplement: Supplementary file 3 — Analysis of outliers. (DOCX 20.9 kb) [file 12885_2017_3335_MOESM3_ESM.docx]

| Appendix Table 1. Analysis of outliers | | |
| --- | --- | --- |
|  | Demographics | Issues leading to delays |
| Case 1  10 day delay, excluding weekends | - Metastatic gastric adenoca. - Procedure: OGD - Indication: Vomiting and constipation | Day -1: “?will need OGD; await CT scan result”  Day 1: “CT shows stent compressed, so OGD requested”  Day 2-7: “Chase OGD” (N.B. New years bank holiday)  Day 7: “D/w gastro; Problem with consultant cover, will need to be done by consultant as procedure complex”.  Day 8: “Still no consultant cover”  Day 11: “Endoscopy mane”.  Day 12: Endoscopy performed.  Key points:  Lack of consultant cover for endoscopy lists |
| Case 2  7 day delay, excluding weekends | - Relapsed squamous cell ca of the oesophagus - Procedure: PEG - Indication: Dysphagia with inadequate nutrition | Admitted with dysphagia  Dietician: PO intake inadequate with supplementation. NG feed as bridge to PEG.  Day 0: “Liase with (gastroenterology consultant) re PEG insertion”  Day 1: UGI CNS: “Waiting for date for PEG; d/w Gastro team”  Day 4/5: “Awaiting PEG”  Day 6: “PEG on Friday (day 9)”  Day 9: PEG inserted  Patient also being reviewed by palliative care for symptom control during this time. Plan: if PEG delayed; for hospice.  Key points:  Palliative patient; valuable days lost towards end of life  Possible lack of communication between oncology and gastro  Different pathway for PEG vs OGD? |
| Case 3  5 day delay, excluding weekends | - Oesophageal cancer - Procedure: OGD - Indication: Dysphagia | Day -2: Admitted with 10/7 dysphagia to liquids and solids. Unable to take meds. Losing weight. Low UO. BNO 5/7.  (NB: OP CT 2/52 previously; circumferential thickening of wall of mid oesophagus. OGD to be arranged by day team.)  Day -1: “Arrange endoscopy in view of complete dysphagia. TPN today”. Nutrition team WR: “Start TPN”.  Day 0: “OGD requested”  Day 3: Nutrition team WR: “A/w stenting. Continue PN”  Day 5: Nutrition team WR: “Endoscopy cancelled yesterday; re-booked for tomorrow. No entry noted by (gastroenterology consultant); we will contact the team and chase their r/v. Continue PN”.  Day 6: OGD + stent insertion  Key points:  8 days of gastro (nutrition) team involvement did not appear to expedite scope  8 days of TPN, with associated risks |
| Case 4  4 day delay, excluding weekends | - Duodenal adenocarcinoma. - Procedure: OGD - Indication: Anaemia (Hb 8.3) and ? malaena | Admitted with abdominal pain, vomiting and ?malaena. Hb 8.3.  Admission plan: “for OGD”. CT abdomen booked.  Day 0: “OGD requested”  “Gastro SpR advised to wait until results of CT until deciding whether to perform OGD or colonsopy”  Day 1: “Gastro SpR advised discussing images at Gastro MDT (4 days later) because pt also required colonoscopy, and because no further bleeding and Hb stable”.  Day 4: Onc WR: “Go for colonoscopy and if no bleeding point found; proceed to OGD”.  Day 5: Colonoscopy done  Day 6: OGD done  Key points:  Clinical decision to delay because no further bleeding and Hb stable  Appropriate delay; waiting for CT and waiting for multi-disciplinary meeting. |
| Case 5  3 day delay, excluding weekends | - Metastatic adenocarcinoma of presumed lung primary. - Procedure: OGD - Indication: Anaemia | Day 0: Admitted with Hb 4.6 and black stools; “? UGI bleed secondary to NSAIDs. D/w Gastro SpR; agrees for OGD mane"  *N.B. At this point, diagnosis was adenocarcinoma of unknown primary.*  Day 1: “OGD cancelled because 2 actively bleeding patients scoped. To call endoscopy mane”.  Day 2: SHO d/w OGD; “Not today. Next slot Monday 17.30. Allowed to go on home leave as Hb stable.  Day 4: “Hb stable. A/w OGD”  Day 5: OGD- normal  Key points:  Short delay excluding weekend  However, the indication was urgent so it was inappropriate for the procedure to be delayed over the weekend.  Gastro SpR engaged in discussions early |
